# Determining Monkey Free Choice Long before the Choice Is Made: The Principal Role of Prefrontal Neurons Involved in Both Decision and Motor Processes

**DOI:** 10.3389/fncir.2016.00075

**Published:** 2016-09-22

**Authors:** Encarni Marcos, Aldo Genovesio

**Affiliations:** Department of Physiology and Pharmacology, Sapienza University of RomeRome, Italy

**Keywords:** prefrontal, monkeys, decision making, neurophysiology, strategy, bias

## Abstract

When choices are made freely, they might emerge from pre-existing neural activity. However, whether neurons in the prefrontal cortex (PF) show this anticipatory effect and, if so, in which part of the process they are involved is still debated. To answer this question, we studied PF activity in monkeys while they performed a strategy task. In this task when the stimulus changed from the previous trial, the monkeys had to shift their response to one of two spatial goals, excluding the one that had been previously selected. Under this free-choice condition, the prestimulus activity of the same neurons that are involved in decision and motor processes predicted future choices. These neurons developed the same goal preferences during the prestimulus presentation as they did later in the decision phase. In contrast, the same effect was not observed in motor-only neurons and it was present but weaker in decision-only neurons. Overall, our results suggest that the PF neuronal activity predicts upcoming actions mainly through the decision-making network that integrate in time decision and motor task aspects.

## Introduction

Decision-making has been traditionally studied using experimental paradigms in which the correct choice is determined by a set of instructions. However, little attention has been paid to how decisions are made when subjects are free to decide when to act or which choice to make. In the latter situation of free choice, most experiments have focused on cases in which the options that are provided are neither correct nor incorrect but have different associated values (Thorndike, 1898; Herrnstein, 1961). Under such conditions, decisions are the result of a noisy, deliberative process in which the value of each option is compared and assessed (Gold and Shadlen, 2007). But what determines what we decide when the options have comparable values?

One possible answer might be provided by the neural activity that precedes the decision-making process (Libet et al., 1983; Haynes et al., 2007; Soon et al., 2008). In a recent neuroimaging experiment, Soon et al. (2008) demonstrated that from brain activity, it is possible to predict future decisions long before subjects report that the decision has reached awareness. In neurophysiological studies with primates, the influence of the previous state of the brain on future choices has been examined using perceptual discrimination and value-based tasks. For instance, during perceptual judgment tasks with ambiguous or nearly ambiguous stimuli, certain neurons in the lateral intraparietal cortex (LIP), medial superior temporal area (MST) and superior colliculus (SC; Basso and Wurtz, 1998; Shadlen and Newsome, 2001; Williams et al., 2003) develop an anticipatory response that is predictive of future choices, even before perceptual evidence is available. Although this type of anticipatory neural activity can help to decide which option to choose, for certain occasions, it needs to be overcome. Neurons from the thalamus are involved in this process (Minamimoto et al., 2014). Other neurophysiological studies have adopted value-based tasks in which the potential options are drawn from a well-known set and have different associated values. Several studies have shown that in these paradigms, decisions can be predicted even before the two options are presented from the anticipatory activity of neurons in the frontal eye field (FEF), supplementary eye field (SEF) and caudate nucleus (CD; Coe et al., 2002; Lauwereyns et al., 2002; Ding and Hikosaka, 2006). Although these reports consistently indicate the presence of anticipatory activity in areas that are predictive of future choice, there are contrasting results in neurophysiology. While some previous neurophysiological studies have found some evidence (Maoz et al., 2013) others have failed to provide similar evidence for the prefrontal cortex (PF; Kim and Shadlen, 1999; Katsuki et al., 2014), notwithstanding its function in goal-encoding (Tanji and Hoshi, 2001; Mushiake et al., 2006; Genovesio et al., 2008, 2014a,b; Yamagata et al., 2012; Genovesio and Ferraina, 2014; Falcone et al., 2015; Stoianov et al., 2016), its activation during free-choice tasks in humans (Rowe et al., 2005; Thimm et al., 2012) and the possibility of biasing target selection by electrical stimulation (Opris et al., 2005). The latter suggests that PF activity during and, likely, before presentation of a stimulus influences future choices when the correct choice is not dictated by external instructions or rules.

**Figure 1:**
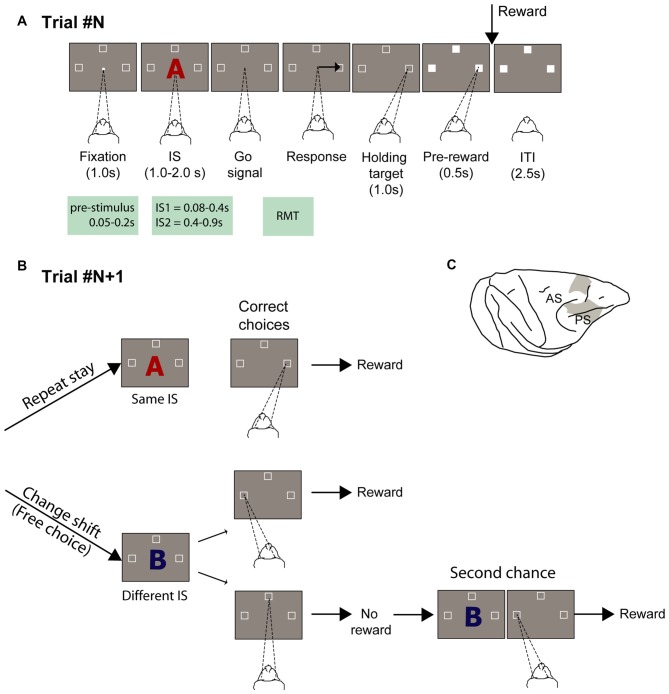
**Experimental task and recording locations. (A)** Temporal sequence of task events. White dot represents the fixation spot, white squares indicate the target location (not to scale), and “A” represents the stimulus (2 superimposed ASCII characters were presented instead). The empty target squares became white after the monkeys selected one of them. The disappearance of the stimulus instructed the monkeys to report their decision with a saccade movement toward one target. After maintaining target fixation for 1.0 s during the target hold period and 0.5 s during the pre-reward period, the reward was delivered when appropriate. Abbreviations: IS1, early instruction stimulus; IS2 late instruction stimulus; RMT, reaction movement time.** (B)** Strategy task. (1) Repeat-stay trials and (2) change-shift trials, also designed as free-choice trials, because the monkeys could freely choose one of two potential targets (left and top goals in the example). The trials in **(B)** are considered example trials following the trial represented in **(A)**, in which the instruction stimulus (IS) was the red “A” and the goal chosen was on the right. The monkeys were required to evaluate whether the IS was repeated from the previous trial: when it was repeated, the monkeys’ task was to choose the same goal as that selected in the previous trial (repeat-stay trials) or shift goals when the stimulus changed (change-shift trials). Repeat-stay trials were always rewarded, whereas shift trials were rewarded half of the time. Second-chance trials followed unrewarded change-shift trials. In these trials, the monkeys had a second chance to respond and be rewarded by choosing the goal selected least recently (left in the example). **(C)** Recordings sites. Abbreviations: AS, arcuate sulcus; PS, principal sulcus.

In this study, we examined the relationship between the activity of neurons in the PF before potential choices are revealed and the choices that are freely made afterward. We identified the group of neurons that were involved in free-choice decision-making and during motor selection and then determined whether the neurons conveyed any prestimulus activity that might bias future choices. To this end, we used a previous dataset of a strategy paradigm that included free-choice trials (Figures 1A,B; Genovesio et al., 2005, 2006). Briefly, the strategy task required monkeys to use a repeat-stay or and change-shift strategy. In each trial, three spatial goals were presented. Based on a comparison between the current instructed stimulus (IS) and that in the previous trial, the monkeys had to select the same spatial goal as in the previous trial (repeat-stay strategy) when the stimulus was repeated or reject the previous spatial goal and select one of two other goals when the stimulus changed (change-shift trials). In change-shift trials, after presentation of the IS, the monkeys faced a decision between options of comparable value. In each of these trials, only one goal led to the reward, which was randomly decided by the computer beforehand. A second-chance trial followed non rewarded change-shift trials in which the monkeys were required to choose the location of the alternative goal to be rewarded (see “Materials and Methods” Section). In this task the monkeys could not commit to a decision before the stimulus appearance because one third of the trials, when the strategy to apply was the repeat-stay trial, were forced choice trials. We considered the change-shift trials as a free-choice condition, because the decision was never dictated by any external instruction or rule as in value-based studies and primarily because, in contrast to previous studies (Barraclough et al., 2004; Padoa-Schioppa and Assad, 2006; Kennerley et al., 2009), the two alternatives could never be compared, based on any perceptual or value-based metric. Thus, the degree of freedom in the decision-making process was maximized.

## Materials and Methods

### Behavioral Task

Two male rhesus monkeys (*Macaca mulatta*; 7.7 and 8.8 Kg) performed a strategy task (Figures 1A,B). All procedures were in accordance with the *Guide for the Care and Use of Laboratory Animals* (1996, ISBN 0-309-05377-3) and were approved by the National Institute of Mental Health (NIMH) Animal Care and Use Committee (IACUC). Details on the experimental procedures have been described in Genovesio et al. (2005, 2006). In brief, the oculomotor task consisted of two types of trials: repeat-stay and change-shift. Each trial started with a central stimulus (0.7° white circle) appearing at the center of a screen. Once the monkey fixated on the central spot, three potential goal locations appeared (2.2° unfilled white squares), 14° up, left and right from the central stimulus. Each stimulus comprised two superimposed ASCII characters, usually in different colors. The monkeys were required to fixate on the central stimulus (±7.5°) for 1 s. Then, the central stimulus was replaced by a visual IS that was presented for 1.0, 1.5, or 2.0 s (pseudorandomly selected). After the IS disappeared, the monkey was required to make a saccade toward one of the three spatial goals within 2 s and maintain its fixation (±6.7°) for 1.0 s. An intertrial time of 2.5 s separated the end of a trial from the beginning of the next one. The sequence of the task events is illustrated in Figure 1A.

The correct choice depend on the strategy that was required, which was to select the same spatial goal as in the previous trial when the IS was the same as in the previous trial (repeat-stay trial) or to choose one of the other two goal locations (change-shift trial or free-choice trial) when the stimulus changed (Figure 1B). Because each stimulus was selected pseudorandomly from a set of three stimuli, 67% of trials were free-choice trials and 33% of them were repeat-stay trials. After a correct strategy response, repeat-stay trials were always rewarded, whereas free-choice trials were rewarded in 50% of cases. Maintaining this reward rate in the free-choice trials precluded the possibility to develop any consistent relationship between stimuli and choices (see Figure 1D in Genovesio et al., 2005).

Unrewarded trials were followed by a second-chance trial, keeping the distribution of the reward rates of each goal comparable and independent of any preference by the monkeys. In the second-chance trial, the monkeys were required to select the goal that remained after eliminating the most recent ones. In all cases, the reward consisted of a 0.1-ml drop of fluid.

### Surgery

In an aseptic surgical procedure and using isoflurane anesthesia (1 to 3%), we performed a 27 × 36-mm craniotomy over the right frontal lobe in each monkey. Then, we implanted several titanium bone screws into the surrounding bone, to which we attached a recording chamber and a head-restraint device with methacrylate acrylic cement. Postoperative analgesia was given for 3–5 days.

### Data Collection Methods

The monkeys’ eye position recorded and monitored with an infrared oculometer (Bouis Instruments, Karlsruhe, Germany) at 500 or 1000 Hz. Single-unit potentials were isolated with quartz-insulated platinum-iridium electrodes (impedance, 0.5–1.5 M at 1 kHz), advanced into the cortex by a 16-electrode microdrive with independent control of each electrode (Thomas Recording, Giessen, Germany). The signal was amplified and discriminated using a multispike detector (Alpha-Omega Engineering, Nazareth, Israel) or a multichannel acquisition processor (Plexon, Dallas, TX, USA). With the latter, neuronal waveforms were always resorted with the Offline Sorter (Plexon). We used CORTEX^1^ to control behavior and collect data. Figure 1C shows the recorded locations in the dorsolateral PF (PFdl) and dorsomedial PF (PFdm; spanning areas 6, 8, and 9).

### Data Analysis

#### Neural Analyses

From the initial dataset on 1456 neurons in the PF (Genovesio et al., 2005), we selected neurons that had a mean activity of at least 1 spike/s within 200–800 ms from presentation of the stimulus (*n* = 887/1456). In this subset, we identified decision neurons and motor neurons. The decision neurons were defined as the neurons that were spatial goal-selective (top, left, right) in the IS1 (early instruction stimulus period) period of the free-choice trials (*n* = 143/887, recorded from 93 experimental sessions; one-way ANOVA, *p* < 0.05). The motor neurons were defined as the neurons encoding the goal location in the reaction and movement time (RMT; *n* = 145/887; one-way ANOVA, *p* < 0.05). Bias activity was studied in the period between 0 and 200 ms before stimulus presentation (prestimulus period). We used this temporal window, because it is the period just before the presentation of the stimulus. In all cases, only correct trials were considered for analyses.

The average firing rate of the neural population was plotted using the mean of the individual neural responses, calculated with a window of 50 ms and a sliding window of 5 ms to smooth the curves. The statistical significance (paired *t*-test) between preferred and nonpreferred goals conditions was calculated using a nonoverlapping window of 100 ms. Only the time intervals in which the neural activity was significantly different for at least three bins are reported. Different windows sizes led to equivalent results (data not shown). The preferred and nonpreferred goals for each neuron corresponded to the goal that was associated with the maximum average activity and the average of the two remaining ones, respectively.

#### Behavioral Prediction

To determine the percentage of choices that could be predicted by the neural activity in the prestimulus period, we implemented a classification procedure with neuron-dropping analysis (Foffani and Moxon, 2004; Lebedev et al., 2004) using neural activity as the predictor variable. We sorted the free-choice trials by neuron and selected goal and calculated the mean firing rate in each trial in the periods of interest. Thus, each neuron had a distribution of mean firing rates for the left, top and right choices in free-choice trials. To classify a trial, we randomly took a trial from each neuron (test trial), and for each selected goal, we calculated a look-up table, consisting of the mean firing rates of all remaining trials (neural response templates). Then, the Euclidean distance between the test trial and templates was used as a criterion to estimate the choice. The trial was classified as belonging to a specific goal location, based on the smallest sum of calculated distances. The neuron-dropping analysis comprised randomly eliminating one neuron in each iteration; thus, the estimate first considers all neurons from a group and the number of neurons decreases until one remains. This procedure was repeated 1000 times to estimate the probability of correctly predicting the future choice for each specific subset of neurons. Increasing the number of iterations did not significantly influence the results (data not shown).

#### Histological Analysis

Toward the end of the data collection, we created electrolytic lesions (15 A for 10 s, anodal current) in locations at two depths per penetration. After approximately 10 days, the animal was deeply anesthetized and perfused with formaldehyde-containing fixative. The brains were later sectioned in the coronal plane and Nissl-stained for cytoarchitectonic analysis (Genovesio et al., 2005). We plotted the surface projections of the recording sites by reference to the recovered electrolytic lesions and the marking pins that were inserted during the perfusion.

## Results

### Neural Response Bias

Overall, the monkeys performed well on the task—Monkeys 1 and 2 had a correct response rate of 96% and 83% in the change-shift trials, respectively, compared with 92% and 88% during the repeat-stay trials. The RMT during free-choice trials was 442.7 ± 7.6 ms for Monkey 1 and 441.4 ± 8.1 ms for Monkey 2.

**Figure 2:**
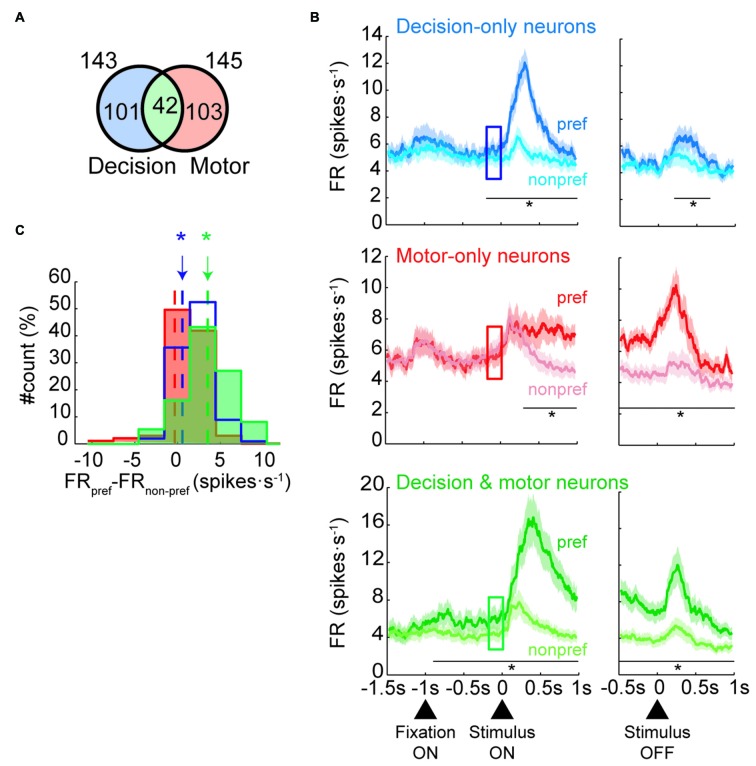
**Neural classification and population activities in free-choice trials. (A)** Number of neurons classified as decision-only, motor-only or decision and motor neurons (not to scale).** (B)** Mean firing rate of each neural population during free-trials. Population activity of decision-only neurons (*n* = 101; Top panel), motor-only neurons (*n* = 103; Middle panel) and decision and motor neurons (*n* = 42; Bottom panel) divided by preferred (dark colors) and nonpreferred goal locations (light colors) of the neurons aligned to the stimulus presentation (left) and offset (right). The squares indicate the prestimulus period (from 200 to 0 ms before stimulus onset; in all panels, paired *t*-test, *p < 0.01). Shaded areas indicate SEM. **(C)** Distribution of the individual difference in firing rate between preferred and nonpreferred goal locations during the prestimulus period (indicated by the squares in **B**) for the three group of neurons (pared *t*-test, *p < 0.01). Dot lines indicates the mean of each distribution.

To determine whether neurons modulated their activity before the stimulus was presented, we focused our analyses on the change-shift trials and on three kinds of neurons: those that were involved in the decision process but not in motor selection (decision-only neurons), those that were involved during motor selection but not during decision making (motor-only neurons) and those that were involved in both decision making and motor selection (decision and motor neurons). Henceforth, the change-shift trials are referred to as free-choice trials, because in these cases, the monkeys were free to choose between two goals without knowing which goal would be rewarded. The decision neurons were defined as those that encoded the future goal location in the first 80–400 ms of the IS (IS1; prestimulus period) whereas the motor neurons are those that encoded it during the RMT period. The prestimulus period corresponded to the time during which the monkeys were first informed on the two potential goals during the free-choice trials. To identify both group of neurons, we performed a one-way ANOVA (*p* < 0.05) with future goal location (top, right, left) as a factor. We identified 143 of 887 (16%) neurons modulated by goal location during the prestimulus period and 145 neurons (16%) modulated during the RMT period with 42 neurons belonging to the two groups (Figure 2A).

Next, we examined whether the population of decision-only neurons showed a prestimulus neural response bias by comparing their activity for choices toward the preferred and nonpreferred goal locations before presentation of the IS. Top panel of Figure 2B shows that the decision-only neurons that were selective for a specific goal location were goal-selective during IS1, as expected. Interestingly, the neurons developed significant differences in the activity between preferred and nonpreferred future goals before presentation of the IS, indicating the presence of a neural response bias. The activity that was associated with the preferred and nonpreferred goal locations began to differ significantly approximately 200 ms (paired *t*-test, *p* < 0.01) before presentation of the stimulus. The activity of these neurons was also significant for 400 ms after the disappearance of the IS (from 200 to 600 ms after IS offset). On the contrary, the activity between preferred and nonpreferred goals of motor-only neurons was significantly different 200 ms after the appearance of IS but not before (middle panel, Figure 2B). Moreover, the difference between conditions persisted during the entire RMT period. The difference between preferred and nonpreferred conditions of the decision and motor neurons is shown in the bottom panel of Figure 2B. The activity between conditions started to significantly differ 900 ms before the IS presentation and remained significant during the entire IS and RMT periods. Figure 2C shows a histogram with the difference between preferred and nonpreferred conditions for each neural group during the prestimulus period. The difference is significant for decision-only and decision and motor neurons but not for motor-only neurons. Moreover, this result held when we selected the half of decision and motor neurons with the lowest maximum selectivity in the IS1 period (paired *t*-test, *p* < 0.05). Thus, we can rule out the possibility that the differences in the prestimulus activity between the groups could be accounted by differences in the degree of selectivity.

**Figure 3:**
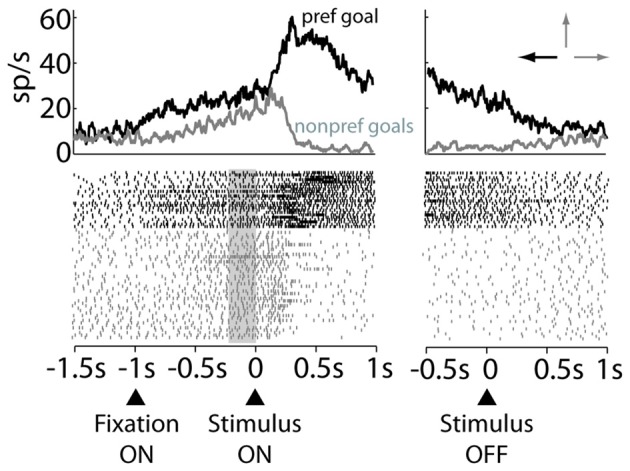
**Raster plot of an example decision and motor neuron.** Trials are sorted by preferred (black) or nonpreferred (gray) goal choice. The data are aligned to the stimulus onset (*Left panel*) and stimulus offset (*Right panel*). Arrows on top of the right panel indicate the preferred (black) and nonpreferred goals (gray). Gray rectangle marks the prestimulus period.

An example of a decision and motor neuron that was selective for the left goal in the free-choice trials is shown in Figure 3. This neuron had higher activity in the decision period (left panel, Figure 3) for the left goal (preferred) than for the top and right goals (nonpreferred goals). Further, it developed a response bias for the future goal choice in the prestimulus period, anticipating the same preference for the left goal, showed during the decision period. Thus, this preference appeared, even before the stimulus was presented and, therefore, before the two alternative goals could be identified. The same preference for the left goal was maintained later by this neuron in the RMT period, declining only after acquisition of the goal (right panel, Figure 3).

### Neural Selectivity and Prediction

**Figure 4:**
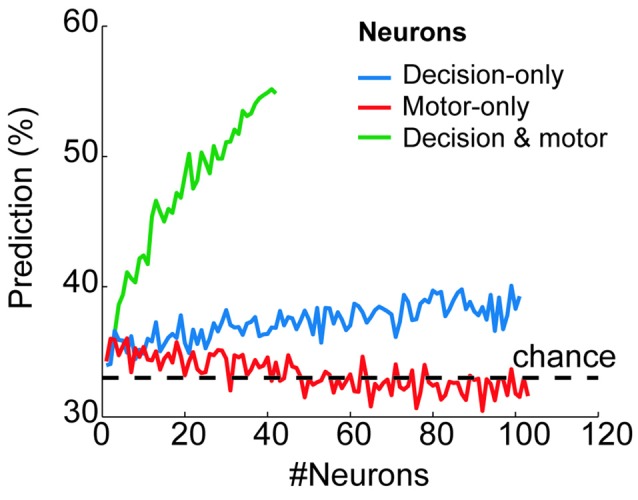
**Behavioral prediction from the prestimulus neural activity of each neural group.** Proportion of trials in which the goal choice was predicted from the prestimulus activity of the decision-only neurons (blue), motor-only neurons (red) and decision and motor neurons (green) when considering a variable number of neurons (from *n* = 1 to *n* = 101, from *n* = 1 to 103 and from *n* = 1 to *n* = 42, respectively). The horizontal dashed line shows the chance level of the prediction (33%).

To quantify how reliably the prestimulus activity of each group of neurons predicted the final goal choice, we used a classification algorithm with neuron-dropping analysis (Laubach et al., 2000; Foffani and Moxon, 2004; see “Materials and Methods” Section). Figure 4 shows the probability of correctly predicting a monkey’s future goal choice as a function of the number of neurons that were used for prediction (from *n* = 1 to *n* = 101 for decision-only neurons; from *n* = 1 to *n* = 103 for motor-only neurons; from *n* = 1 to *n* = 42 for decision and motor neurons). The prediction exceeded chance values (33%) for decision-only and decision and motor neurons, increasing in both cases with the number of neurons that were used together. Chance value was 33% because in the prestimulus period, before IS presentation, all three targets were potential goals. On the contrary, the prediction remained at chance level for the motor-only neurons, independently of the number of neurons used for prediction. Importantly, the decision and motor neurons showed a much greater proportion of correctly predicted trials compared with the decision-only neurons. Moreover, the prediction from the prestimulus activity of decision and motor neurons peaked at ~55%, well above chance and close to the maximum percentage of trials that could be predicted (66%) considering that before the presentation of the stimulus the potential choices were three. These results show a high predictive power of the future choice of the prestimulus activity of decision and motor neurons. Moreover, the prediction is notably higher than that provided by the decision-only neurons.

## Discussion

In this study, we have provided evidence that pre-exiting activity of some neurons in the PF biases monkeys’ future choices. In particular, we have shown that the prestimulus activity of the decision and motor neurons had a high predictive power during free-choice trials reaching a value of 55% (chance value is 33%) when all neurons of the group were pooled. We differentiated motor and decision activity based on the period of the analysis, notwithstanding the fact that other functions, such as attention to target and motor vs. abstract goal coding, could not be dissociated. Our central finding is that a preference for the same target emerged in these neurons before presentation of the stimulus, indicating that the same future choice is encoded already in the pre-existing activity. Moreover, the neural activity bias emerged 900 ms, but not earlier, before presentation of the stimulus, suggesting that the neural response bias originated only after the beginning of the trial, when the monkeys were engaged in the new trial. However, they did not yet commit to any decision as shown by the good performance in the repeat-stay trials that imposed a forced choice (see Figure 2 in Genovesio et al., 2005).

To achieve a high level of accuracy in the strategy task the monkeys needed to perform several cognitive operations in addition to a free choice. First, they needed to maintain in memory the previous IS and chosen goal to be able to select the correct goal in the trial. Second, they needed to decide to either stay or switch to a different response based on the comparison between IS in the previous and current trial. Last and only in the free-choice trials they needed to choose one of two targets. While the other cognitive operations were examined by our previous articles (Genovesio et al., 2005, 2006, 2008) here we focus only on the free-choice. Free-choice trials incorporated at least two task features that provided a suitable framework to study modulations in anticipatory neural responses. One important feature that differentiates our study from most existing neurophysiological reports is that the two alternative goal locations in each free-choice trial were not specified before the stimulus was presented; thus, the monkeys could never covertly choose a specific goal or prepare for a specific action. This uncertainty depended on the task design, which interleaved the free-choice trials with repeat-stay trials in each block. Hence, any anticipatory activity that biased the goal choice appeared before the alternative goals were known. The second hallmark of the free-choice trials was that the two goals never differed in value in terms of reward probability. The presence of rewarded second-chance trials after unrewarded change-shift trials maintained a comparable number of rewards that were associated with each goal location, independent of the monkeys’ past choices. By balancing the goal values, the influence of pre-existing bias activity on the decision process might have increased and thus become detectable, in contrast with previous studies. Indeed, it has been proposed that any small bias can unbalance the competition between population of task selective neurons (Rolls and Deco, 2011; Marcos et al., 2013). Our task design and those of other studies share the feature that the neural response bias is not accounted for by the motivation to anticipate the delivery of a reward due to a delay between presentation of the stimulus and the go signal.

Many studies have examined the function of PF in decision-making, most of which have focused on tasks in which the correct response depends on the comparison between sensorial events using disparate sensory systems and domains, demonstrating how PF cells participate in decision-making (Kim and Shadlen, 1999; Hoshi et al., 2000; Freedman et al., 2001; Brody et al., 2003; Genovesio et al., 2009, 2011, 2012, 2015; Hussar and Pasternak, 2013; Roy et al., 2014; Marcos et al., 2016). However, few studies have explored the influence that any pre-existing activity of PF neurons has on decision-making, and moreover, they seem to have contradictory results.

Several neurophysiological studies have failed to observe an anticipatory response bias in PF neurons when monkeys perform perceptual discrimination tasks, even when the psychological judgments were difficult (Kim and Shadlen, 1999; Katsuki et al., 2014). In Katsuki et al. (2014), for example, monkeys were required to locate a salient stimulus that was surrounded by other distractors and release a lever when a subsequent stimulus was presented at the same location as the salient stimulus. They found that activity of the parietal area LIP but not of the PFdl before presentation of the stimulus was predictive of the monkeys’ future decisions regarding the presence of the salient stimulus, although only transiently. In a recent categorization experiment with ambiguous stimuli, Roy et al. (2014) identified neurons in the PF that categorically represented ambiguous stimuli, but the categorical presence emerged only after presentation of the stimulus and only with a longer latency than with unambiguous stimuli. One possible explanation for the absence of a neural bias in the PF in these experiments (Kim and Shadlen, 1999; Roy et al., 2014) is that they required a perceptual choice rather than a goal choice as in our task. In a recent study (Mochizuki and Funahashi, 2016), it was reported that the prestimulus activity of some neurons in PF was predictive of the future choice of monkeys. However, whether the neurons that exhibited the predictive power were those involved in decision or motor processes was not distinguished.

Conversely, using value-based free-choice decisions, a recent neurophysiological study reported anticipatory activity of neurons in the PFdl that was predictive of future choice (Maoz et al., 2013), raising the possibility that value-based decisions, as opposed to perceptual decisions, can be influenced by activity bias in the PFdl. In Maoz et al. (2013), the task had two goals with different associated values, and the upcoming choice could be predicted from the anticipatory activity when the two values were alike, similar to the modulation that is observed in neurons in the LIP, MST and SC during perceptual tasks (Basso and Wurtz, 1998; Shadlen and Newsome, 2001; Williams et al., 2003). In contrast to our study, they found that the neurons that are involved in the decision process are not the same as those that predicted the upcoming choice before presentation of the stimulus. This result led the authors to hypothesize the separation between a network of neurons with predeliberate spontaneous activity that biases future choices under free-choice conditions and a network of neurons that mediate rational decision-making.

Unlike in earlier studies, our task has the advantage of not requiring any comparison between alternatives to determine the correct goal location, as in the perceptual tasks, or any evaluation and comparison of the values of the alternatives, as in value-based decision tasks. Under these new conditions, we found that neurons involved in goal coding from the decision to the motor phase, in the PF showed prestimulus activity that was highly predictive of future decisions. Importantly the decision neurons’ activity could not depend on differences in visual responses because all three targets were always presented. Thus, our results indicate that neurons that exhibit a bias in the prestimulus period are not necessarily dissociated from those that are involved in decision formation, as proposed (Maoz et al., 2013), and that a neural response bias can emerge, even in the absence of a value-based decision in line with the results of Mochizuki and Funahashi (2016). In our free-choice condition, decision and motor neurons anticipated the choice long before the stimulus was presented and held it until the onset of movement. One explanation for these differences is that the task in Maoz et al. (2013) required a comparison between alternatives, whereas it was unnecessary to compare any feature of the alternative goals in our task. In Maoz et al. (2013), the comparison might have interfered with the prestimulus neural response bias during the decision period, which did not occur in our case.

In earlier studies (Genovesio et al., 2005, 2006; Tsujimoto et al., 2008), we have shown that PF cells contribute to the implementation of the strategy from stimulus identification to the goal choice. Although these neural representations reflect the strategy implementation, they could not account for the goal selection in the free-choice condition, which remained unexplained. Our study increases our understanding of the function of the PF in the computations involved in the goal choice. It shows that the activity of the decision and motor neurons and, only to some extent, the activity of decision-only neurons but not that of motor-only neurons is not limited to the decision and motor processes but emerges before presentation of the stimulus, biasing the upcoming choice.

In conclusion, the pre-existing activity of neurons in the PF contributes to the decision process in free-choice conditions but we cannot rule out the contribution of other areas as the source of the bias. Further, our study has examined the prefrontal contribution of the PFdl and PFdm to free choice at the single-cell level, showing that under no external constraints or instructions, the pre-existing state of the decision and motor PF neurons that appear to bridge in time the decision and motor phases (Fuster, 1990) has a significant impact on the upcoming choice.

## Author Contributions

AG performed the experiment, EM analyzed the data and AG and EM wrote the article.

## Conflict of Interest Statement

The authors declare that the research was conducted in the absence of any commercial or financial relationships that could be construed as a potential conflict of interest.

## Footnotes

^1^http://dally.nimh.nih.gov
